# A Study Protocol for Safeguards Child and Adolescent Mental Health Rapid Response Teams (‘Safeguards Teams’) Service

**DOI:** 10.5334/ijic.7004

**Published:** 2023-08-10

**Authors:** Valsamma Eapen, Brigitte Gerstl, Teresa Winata, Rajeev Jairam, Giles Barton, Michael Bowden

**Affiliations:** 1 Academic Unit of Infant, Child and Adolescent Psychiatry Services (AUCS), SWSLHD and Ingham Institute, Australia; 2 Discipline of Psychiatry and Mental Health, University of New South Wales, Sydney, Australia; 3 Infant, Child and Adolescent Mental Health Service (ICAMHS), South Western Sydney Local Health District, Sydney Australia; 4 School of Medicine, Western Sydney University, Sydney, Australia; 5 Mental Health Branch, NSW Ministry of Health, Sydney, Australia; 6 Sydney Children’s Hospitals Network, Sydney, Australia; 7 Discipline of Psychiatry, Sydney Medical School, University of Sydney, Sydney, Australia

**Keywords:** mental health services, children and adolescent mental health services, models of care, early intervention, psychological distress, crisis intervention

## Abstract

**Introduction:**

As the number of children and young people (CYP) presenting to Emergency Departments (ED) with acute mental health (MH) presentations has been steadily increasing over the years and further accelerated by the pandemic, there is an urgent need to develop and evaluate innovative solutions to respond to this growing challenge.

**Description:**

The evaluation of the Safeguards Teams Program (STP) aims to ascertain the impact, implementation and economic analysis of this acute rapid response recovery-focused, trauma-informed assessment and brief intervention for CYP (aged 0–17 years) presenting in acute MH crisis and their families/caregivers.

The STP will support consumers (patients) and their families/caregivers to navigate the complex and often fragmented child and adolescent MH services (CAMHS) landscape, thereby avoiding unnecessary ED presentations or hospitalisations, and facilitating comprehensive assessment and appropriate care pathways for those who present in crisis.

**Discussion:**

The STP is expected to provide CYP in MH crisis and their support networks with early access to evidence-based specialist care at the right place and time.

**Conclusion:**

Implementation of the STP will assist with identifying and addressing gaps in acute care for CYP and provide the necessary evidence for service redesign in collaboration with consumers, service providers and other stakeholders.

## (1) Introduction

Around one in seven (15%) children and adolescents globally are diagnosed with mental health (MH) conditions [1]. MH conditions emerge early, with 34.6% of all consumers presenting by the age of 14 years and 62.5% diagnosed by the age of 25 years [2,3]. MH concerns among young people contribute to 45% of the total burden of disease, more specifically among individuals aged 10–24 years (2, 3), with adolescents and young adults reported to have the lowest access to MH care compared to other age groups. Recent Australian data has shown that child and adolescent MH presentations to the Emergency Department (ED) for self-harm or suicidal ideation have been increasing by 8% annually prior to the COVID-19 pandemic but that this has accelerated to 19% since the COVID-19 outbreak [2]. The increase was particularly pronounced in female adolescents with per annum overall increase in 13- to 17-year-olds reaching 47.1% in the post-COVID period from March 2020 to June 2021. Furthermore, a study of hospitalisations and ED presentations to a Children’s hospital post the first wave of the COVID-19 lockdown from June 2020 to February 2021 found a 30–55% increase in health service use for mental health disorders, especially for female adolescents, while that for acute infections and injuries remained persistently lower [4].

As the number of children and young people (CYP) presenting to ED with acute MH presentations increases, there has been an attempt to find alternative solutions to manage crises within the community setting for vulnerable children and young people (CYP). The literature reports varied approaches to specific interventions, such as Multisystemic Therapy for behaviour disorders (4), Assertive Community Treatment for high-risk and difficult to engage adolescents who have a severe mental illness (5), and ‘one-stop shops’ of Integrated Community-Based Youth Service (C-BYS) Hubs that aim to cater for psychosocial, educational, physical and MH needs in an early intervention model (6). However, these models require specific funding, strong collaboration, and integration of services.

The approach to acute crisis presentation requires accessibility and availability outside of usual business hours, outreach into the community, and individually tailored care that is specific for CYP and their families while also ensuring that appropriate referrals and linkages with other relevant services are in place to support CYP consumers following a crisis intervention. Without appropriate post-intervention support, CYP with MH conditions are highly likely to have poor health and social outcomes and high ongoing service user needs. Failing to support CYP during the crisis and adequately supporting them thereafter is expected to have a significant adverse impact on their long-term trajectory that may include poor adult MH, low school engagement and performance, unemployment, high dependence on welfare, unstable housing conditions, involvement with the child protection system, criminal activity, drug and alcohol dependency, and premature death [5].

A recent systematic review found evidence for the effectiveness of crisis interventions for CYP who are in acute MH distress via reducing ED presentations and hospitalisations and reducing health system costs, but the review also highlighted the scarcity of studies in this area and called for further studies to bridge this knowledge gap [6]. Here we detail the Safeguards Teams Program (STP) which comprises of dedicated and skilled child and adolescent acute rapid response team designed to provide innovative and best practice care to CYP aged 0–17 years who are experiencing acute MH distress.

### (1.1) Aims of the Safeguards Teams

The Safeguards Teams Program aims to improve access to timely, evidence-based, recovery-focused, and trauma-informed assessment and brief intervention for children and adolescents presenting in acute MH crisis and their families/caregivers.

### (1.2) Hypothesis and outcomes

The STP will reduce the rates of CYP re-presenting to the ED with acute MH concerns including the use of emergency services and/or necessitating re-hospitalisations and result in better engagement with services (primary outcome), and leading to better mental health outcomes, thereby minimising school (better school attendance) and psychosocial (better engagement and participation) disruption (secondary outcomes).

An additional hypothesis is that the implementation of the STP program will be feasible, acceptable, and accessible and will result in effective referrals and linkages to relevant services post-intervention with the uptake of these recommendations and service satisfaction for consumers (CYP and their families), service providers (clinicians and health managers) and other stakeholders (education, social and welfare services). An economic evaluation will also be undertaken to ascertain the value for money by comparing the incremental cost and benefit based on child and family outcomes and service experiences. Return on investment, associated with wide-scale implementation, will be modelled based on the trial outcomes.

## (2) Description of the Methodology

### (2.1) Study design and setting

The study will utilise a pre- (Time 1/baseline) and post-intervention (Time 2) design to compare outcomes of individuals who receive STP at entry into the program and then on exit from the STP followed by data collection at 3 months post-intervention (Time 3) for re-presentation to ED or in-patient wards including the use of emergency services (e.g., ambulance, police) and engagement with referred services and uptake of recommendations. In addition to questionnaire/survey data collected at the three-time points, EMR data will be collated for those who participated in the STP program and from another CAMHS service site where CYP presenting in acute crisis received care as usual. Further qualitative study using focus groups and in-depth interviews, and ethnographic approaches, such as observations and reflections will be completed with consumers (CYP and their parents/caregivers), service providers and relevant stakeholders. An implementation evaluation of the overall impact, implementation process and economic analysis will also be carried out.

### (2.2) Sample population

The sample for the STP will include children and adolescents 0–17 years who are experiencing an acute MH crisis, who are not currently undertaking MH treatment and would benefit from a rapid response MH service. Services will be provided for both CYP and their parents/caregivers.

### (2.3) Participant inclusion and exclusion criteria

#### (2.3.1) Inclusion criteria

- Children and adolescents aged 0–17 years presenting in an acute mental health crisis (we defined a mental health crisis as an occurrence when a person is faced with an experience that exceeds their ability to cope and where an individual has a need (or perceived need), for an urgent or immediate response) [7].
- Parents/guardians of CYP (0–17 years of age) accessing STP.
- Service providers and stakeholders who have knowledge of or have interacted with the Safeguards Teams.

#### (2.3.2) Exclusion criteria

Child or adolescent is actively engaged in a treatment program and their usual treating practitioner/team is available to see them.

Acute risk of harm to self or others that require immediate intensive/hospital-based specialised care that cannot be provided in the community by the STP.

### (2.4) Control group

We will incorporate a control group to improve the assessment of the potential benefits of the STP intervention. This group will comprise children and adolescents (aged 0–17 years) who visited the emergency department (ED) during the same period as those who received the STP intervention but did not receive the STP intervention. By conducting a comparative analysis of the outcomes between the intervention and control groups, the efficacy of the STP intervention can be evaluated in a more robust manner.

### (2.5) Intervention: Safeguards Teams Program

The STP is an innovative new service that will provide patient-centred outreach and acute crisis intervention for children and adolescents, and their families/caregivers experiencing MH crises, for up to 6 to 8 weeks. Follow-up services via referral and linking to relevant services with ‘warm’ handover will support consumers (patients) and their families/caregivers to navigate the complex and fragmented mental health service landscape, thereby avoiding re-presentations to ED or other acute services including re-hospitalisation. The teams are community-based and will provide rapid, mobile, intensive, and flexible short-term delivery of skilled evidence-based interventions to resolve mental health crises. They will provide extended hours for mental health services and partner with relevant health services to ensure 24/7 support to CYP and their families while in crisis.

The STP will employ a multidisciplinary approach in managing acute and complex MH presentations of CYP who are referred to the service. Complex presentations in this population often require several specialised interventions to manage their presentation and typically include a comprehensive assessment including risk assessment and safety planning, psychiatric reviews, psychological interventions, family therapy and/or other relevant supportive referrals. The multidisciplinary team allows a holistic approach to managing complex risk and MH presentations that often precipitate and maintain crisis presentations. To achieve this, STP will be multidisciplinary and comprised of child psychiatry, senior nursing, and allied health professionals with the clinical expertise to deliver crisis assessment, specialist clinical care and short-term therapeutic interventions for young people with high and complex mental health needs and their families/caregivers. The Teams will grow a much-needed sustainable child and adolescent mental health workforce needed as the STP evolves and support the expansion and professional development of a skilled multidisciplinary child and adolescent workforce.

The Teams will respond to young people in their schools, homes/placements, and communities and in hospital-based settings (EDs/Wards), through face-to-face, phone and telehealth appointments. This flexible model will be adapted to work across rural, regional, and metropolitan locations and be tailored to meet local cultural and diversity needs. Within a staged care approach, the STP will provide a more intensive acute service component (tier 4) on the spectrum of care supporting the work of community-based child and adolescent mental health services (tier 3) and the tertiary acute child and adolescent inpatient units. To manage crisis presentations, the Safeguards Teams will employ a range of evidence-based interventions that are indicated for use with CYP to stabilise their presentations. The Safeguards Teams will provide both CYP-centred interventions as well as family/caregiver-centred care with strategies and intensity of treatment to match the acuity and nature of the presentation. Cognitive Behavioural Therapy, motivational interviewing, risk assessment and management as well as supportive counselling will be utilised to improve MH outcomes for CYP. These interventions along with supportive referrals and a ‘warm’ handover to referred services ensuring uptake of recommendations will be regularly utilised by the STP.

## (3) Service components

The development of local service models contextualised to the setting is expected to utilise the local area service planning processes and incorporate co-design and co-production with consumers, parents/caregivers, staff, and other stakeholders.

The Safeguards Teams are:

- Embedded within and additional to existing Child and Adolescent Mental Health Services (CAMHS) under the common governance of CAMHS service structures.
- Comprised of skilled multidisciplinary clinicians able to provide a range of evidence-based therapeutic interventions
- Available to work within an assertive outreach model and deliver specialist assessment, care coordination, clinical care, and therapeutic interventions
- Flexible and provide care across the 0–17 age range
- Able to ensure that access to care is based on level of acuity, risk, complexity, and distress, and is not limited by diagnosis or co-morbidities such as developmental disability, eating disorders, children in out-of-home care and younger children. Availability of other supports and alternative care options may be considered.

The key principles that support the delivery of holistic, high-quality mental health care via the STP include:

1. Rapid, accessible, and inclusive
2. Any child or adolescent with a mental health crisis regardless of diagnosis or sociodemographic status
3. Child and young person-centred, family/caregiver-centred
4. Developmentally appropriate
5. Recovery-oriented, strengths-based culturally safe and trauma-informed
6. Collaborative, integrated care, service navigation
7. Draws on and contributes to existing evidence, continually improving

### (3.1) Referrals into the STP

**Figure 1:**
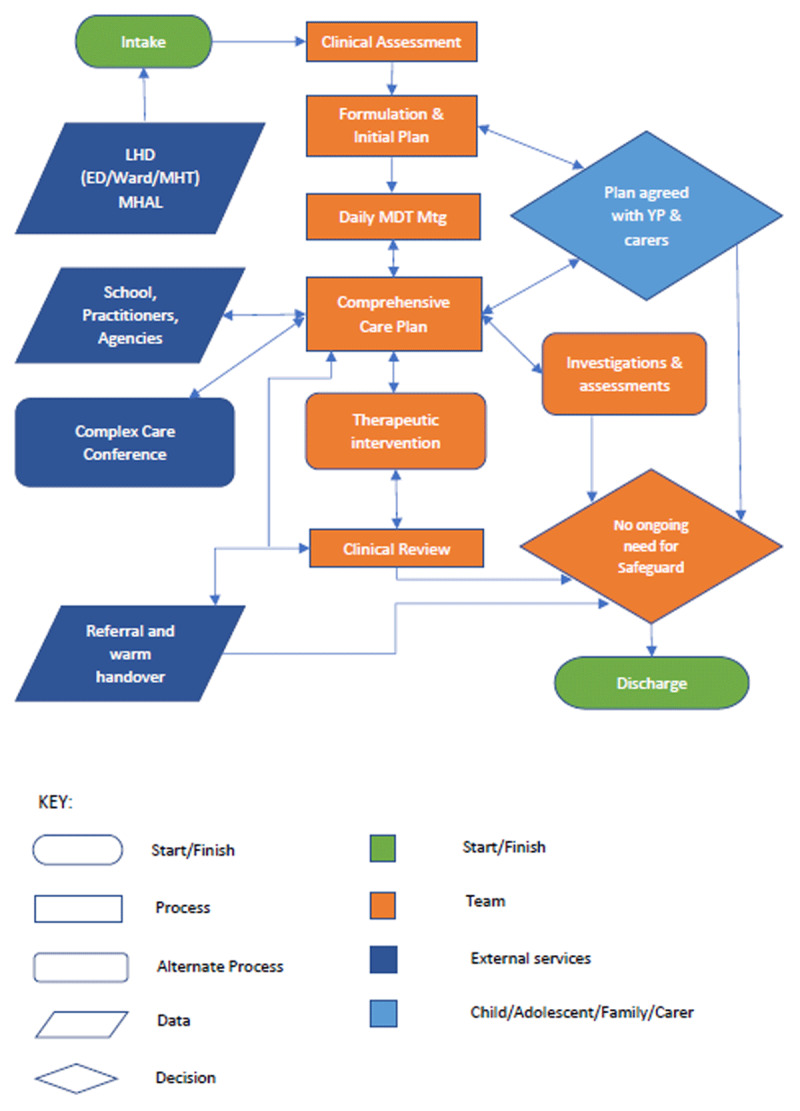
A flow diagram of the processes associated with the Safeguards Teams. * Notes: ED- Emergency Department; LHD- Local Health District; MDT Mtg- Multidisciplinary Team Meeting; MHT- Mental Health Team; MHAL- Mental Health Access Line; YP- Young Person.

The STP is meant to be in addition to and not replace existing, functional crisis response systems already in place. Subject to local unmet needs, the STP can respond to most or all scenarios. Requests for assistance may come from the community including school or other services, via the ED, or by other Community MH teams. Additionally, the STP will work closely with local General Practitioners (GP), schools, and other organisations to route crisis presentations to STP so that the team can do outreach assessments minimising the need to call the police or the ambulance where it is safe to do so. In addition to providing acute care for up to 6 to 8 weeks, consumers will be supported for the next steps before being discharged (Figure 1).

### (3.2) Consent to the evaluation

Consumers who consent to participate will be provided with a link to information, consent, and entry to the STP measures and surveys. In line with the Australian Paediatric Research Ethics & Governance Network [5] and the NHMRC research ethics and governance framework [8], consent will be required from both the child and parent(s)/legal guardian(s). In addition to the quantitative surveys, qualitative data collection will involve CYP and families, service providers and other stakeholders.

The above process will ensure that the participants are well informed about (1) the reasons for conducting the research study, (2) the risks and benefits of participating in the research, (3) what they can expect if they agree to participate, (4) their rights and responsibilities (including their rights to withdraw), (5) provision of voluntary consent to participate (that is, not subject to coercion or inducement), and (6) the ability to withdraw at any stage from the study.

## (4) Data collection

### (4.1) Impact evaluation of the Safeguards Teams Program

The overall program impact will be ascertained as outlined in Table 1.

**Table 1:** Primary and secondary outcomes.


OUTCOME	OUTCOME DOMAIN	PRIMARY SOURCE	SECONDARY SOURCE

Primary	To compare the overall utilization of ED, acute adolescent inpatient and community CAMHS and other acute care services pre and post implementation of the safeguard program.	Cohort study questionnaires and EMR data for the participants of the STP program and EMR data from another CAMHS service site where CYP presenting in acute crisis received ‘care as usual’	EMR data

	Engagement with services	EMR data for STP program and for ‘care as usual’ group	Focus groups/interviews with CYP, parents and service providers; ethnographic observations/reflections

Secondary	Mental health outcomes	Cohort study questionnaires EMR data	Focus groups/interviews with consumers, service providers and stakeholders

	School attendance/social participation		

	Parenting and relationship		

	Service satisfaction		


An analysis of routinely collected electronic medical record (EMR) data of patients and selected pre- and post-discharge health outcome measures/questionnaires will be conducted.

A mixed-methods approach will be employed to examine any improvements regarding access, engagement, uptake/integration of care within mental health services following the implementation of the STP. Sociodemographic variables will be collected at baseline which is known to impact mental health crisis presentations such as the age of the consumer at presentation, gender, culturally and linguistically diverse background of consumers (CALD), Aboriginal and Torres Strait Islander status, who the child is living with including out of home care status, family constitution and socioeconomic status (based on Socioeconomic Index for Areas [SEIFA] index).

Mental health outcomes will be ascertained using pre- (at admission) and post- (at discharge) questionnaire surveys completed by clinicians, CYP and their parents/caregivers (Table 2). Qualitative research methods, involving focus groups and interviews, will be conducted with clients, parents/caregivers, service providers and stakeholders to identify facilitators and barriers to children/young people’s, family members/caregivers’ experiences of engagement, involvement, uptake of mental health services over time. A quantitative satisfaction survey will also be utilized to determine the level of satisfaction experienced by CYP and their families with the STP in providing an integrated experience of mental health care.

**Table 2:** Schedule of quantitative data collection assessments at time points 1, 2 and 3.


Children and adolescents Parent/caregiver	Pediatric Symptom Checklist—Youth Report (Y-PSC) [9] (for CYP: 12–17 years) [10] Pictorial version (6–11 years) [11] Pediatric Symptom Checklist – Parent report [10]

Child (12–17 years) Parent/caregiver	Your Experience of Service (YES) [12] Carer Experience Survey (CES) [13] Kessler Psychological Distress Scale (K10) [14,15]

Clinician	Health of the Nation Outcome Scales for Children and Adolescents (HoNOSCA) [16] Children’s Global Assessment Scale (CGAS) [17] The Clinical Global Impression – Improvement and severity scale (CGI-I & CGI-S) [18]


A semi-structured questionnaire has been developed to gather not only baseline demographic data but also information regarding how CYP functions (information that is not currently collected on a hospital EMR system) relative to mental health service access; attendance, participation and performance at school; participation in sports or other physical or social activities; and the current relationship between the CYP and parent(s), other family members (i.e., siblings), friends, and social support network connections.

Data will be collected at each time point.

#### (4.1.2) Description of the Measurement Tools

*NSW Mental Health Carer Experience Survey* provides a better understanding of the experiences, needs and outcomes of carers. The measure consists of 37-items.

*Children’s Global Assessment Scale (CGAS)* scale is a clinician-rated scale used to rate the functioning of children and young people aged 6–17 years old related to their psychological and social functioning. It is a brief- 10-item observer-rated scale.

*Clinical Global Impression – Severity scale (CGI-S) and Improvement scale (CGI-I)* comprise a brief- 3-item observer-rated scale that requires the clinician to assess the overall severity of the individual’s illness and overall improvement in the individual’s illness relative to a baseline state.

*Health of the Nation Outcome Scales for Children and Adolescents (HoNOSCA)* is a clinician-rated measure. The first section consists of questions related to the different types of problems that a child or adolescent might be experiencing; the second part of the survey relates to the parent/caregiver’s knowledge of the nature of their child’s difficulties and information about MH services available to support the child and the family.

*Kessler Psychological Distress Scale (K10)* explores questions about depressive symptoms that CYP may have experienced. The survey is a 10-item measure and explores distress based on questions about anxiety and depressive symptoms that a person with a MH disorder may experience within a 4-week period.

*Pediatric Symptom Checklist—Youth Report (Y-PSC)* is a 17-item measurement psychosocial screen designed to facilitate the recognition of cognitive, emotional, and behavioural problems completed by youth aged 12–17 years and up. Similarly, the parent-completed Pediatric Symptom Checklist is also a 17-item measurement tool and provides a measure of their children’s psychosocial functioning.

*Pictorial Pediatric Symptom Checklist (PPSC)* is a pictorial psychosocial screen designed to facilitate the recognition of cognitive, emotional, learning, and behavioural problems completed by children and youth aged 6–11 years and up. It is a brief 35-item measurement tool.

*Your Experience of Service (YES)* questionnaire explores the experiences of consumers about both their current and previous use of MH services.

### (4.2) Implementation evaluation of the Safeguards Teams Program

A mix of qualitative, quantitative, and ethnographic approaches will be used to examine the effectiveness of the implementation of the STP with a focus on key implementation outcome metrics including acceptability, appropriateness, fidelity, feasibility, coverage, cost, and sustainability as detailed in Table 4. This includes conducting interviews and focus groups with consumers, mental health clinicians, primary care clinicians, hospital clinicians, government agency representatives, and community managed organisations/stakeholders; conducting observations; questionnaires and benchmarking against national mental health standards, to address: (1). What system and service-based changes have occurred? (2). What are the components of a successful Safeguards Teams model of care?; and most importantly, (3). What are the perceptions and experiences of stakeholders about the transition preparation phase, assessment administration, staff delivery of services, family (re)engagement and readiness of the Safeguards Teams model of care (Table 3)?

**Table 3:** Schedule of qualitative data collection assessments collected at time points 1, 2 and 3.


COHORT	MEASUREMENT NAME

Child (12–17 years) Parent/caregiver Clinician	Qualitative in-depth interviews

Child (12–17 years) Parent/caregiver Clinician	Focus groups

Clinician Healthcare providers and community stakeholders	Ethnographic study – observations and self-reflections


**Table 4:** Implementation evaluation metrics.


MEASURE	QUESTIONS ADDRESSED BY EACH IMPLEMENTATION MEASURE

Acceptability	Do all stakeholders including consumers, caregivers/families, and service providers, perceive the STP as acceptable?

Adoption	To what extent do consumers and service providers utilise/find it easy to use the STP?

Appropriateness	Do stakeholders perceive the STP as relevant and useful?

Fidelity	Is the STP applied as intended? Are all components delivered as planned?

Coverage	Are all eligible service users reached?

Cost	How much does it cost to successfully implement the STP?

Sustainability	What are the factors that will allow STP to be scaled-up and sustained in the long term?


The STP processes of how care pathways, practices and agreements have been established and developed from ED, mental health services and community-managed organisations for children and adolescents with acute mental health crises will also be evaluated using a mixed-methods approach. This includes exploring how clinical practice changes have occurred within ED, mental health, and community-based organisations to prevent or decrease avoidable hospital ED presentations and hospital admissions (e.g., ED avoidance, facilitated linkage and engagement with services). The data collection source will include interviews and focus groups with mental health clinicians, primary care clinicians, hospital clinicians, government agency representatives, community-managed organisations/stakeholders, and ethnographic observations in STP multidisciplinary meetings and stakeholders/steering committee meetings.

#### (4.2.1) Data analysis

*Quantitative analysis*: The effectiveness of the Safeguards Teams program will be evaluated by examining the difference in outcomes on entry before participants enrol into the program, and on exit, following a Safeguards encounter, using appropriate statistical methods for different outcomes. Descriptive analyses will explore changes following Safeguards Team’s service delivery. We will use paired samples t-test to compare pre- versus post-intervention outcomes as per measures outlined earlier. Additionally, percent change at entry and exit from the STP from baseline for efficacy variables, will be analysed using analysis of covariance (ANCOVA) with respective baseline value as covariate. Least-square means (LSM) and 95% confidence intervals will be evaluated from the ANCOVA. The data of percent changes will be assumed as normally distributed.

*Qualitative analysis*: In-depth semi-structured interviews of consumers and service providers (peer-workers and clinicians) will be conducted using the Consolidated Framework for Implementation Research (CFIR) [19] framework to ascertain feasibility, acceptability, appropriateness, and sustainability for both children and adolescents (12–17 years) and adults (≥18 years) who were involved in the program. Audio recordings will be transcribed using MS Word, imported into NVivo™ version 12 [20] for data management and grouping, and thematically analysed. Braun and Clarke’s six-phase method of thematic analysis will be implemented to ensure rigour in the analysis process [21].

*Economic Evaluation:* The economic evaluation will aim to understand the cost-effectiveness of the Safeguards Program to inform future decision-making. Trial-based economic analyses will be conducted to ascertain value for money by comparing (i) the incremental cost associated with the STP, including implementation costs, and (ii) incremental benefit assessed according to patient-reported outcome and experience measures and clinical measures. Return-on-investment, associated with wide-scale implementation, will be modelled based on the trial outcomes. Dynamic Simulation Models (DSMs) will be used to identify optimal combinations of services and referral trajectories that will provide policymakers and clinicians with practice-specific information to inform clinical decisions and forecast the potential impacts of services on outcomes. The evaluation will consider the economic return on investment as well as social return on investment.

#### (4.2.2) Ethics and dissemination of results

The study has been approved by the University of New South Wales Human Research Ethics Committee, reference HC220192, and SWSLHD Human Research Ethics and Governance, reference 2022/ETH00808.

## (5) Discussion

In most cases, little is known about the relative effectiveness and efficiency of different CAMHS delivery models [19]. One reason for this is a paucity of outcome and activity data available for CYP with acute MH conditions. Hence, there is an urgent need for specialist child and adolescent acute response MH teams. Outcomes associated with the implementation of the STP will assist with identifying gaps in acute care for CYP in the community and provide opportunities for alternative routes of MH care, to shift reliance from ED or inpatient services. Schley and colleagues [22] reported on the Victorian IMYOS in Australia and described outcomes on 44 high-risk, difficult to engage youth. They demonstrated that a timely and individual-centric collaborative outreach approach is associated with positive treatment outcomes and engagement of CYP who are at high risk to themselves or others and who have been unable to engage with other treatment services. Similarly, a systematic review exploring ‘Assertive Community Treatment’ for youth with severe mental illness who were reported to be difficult to reach, demonstrated positive effects in terms of psychiatric symptom improvement and reduced frequency and duration of psychiatric hospitalisation [23].

It is expected that the STP will provide CYP with MH conditions and their support networks with early access to comprehensive assessment and evidence-based acute response and referral and linkages to appropriate care delivered by specialist clinicians and support services. Outcomes from the STP may also provide clinicians involved in CAMHS with: (1) a more robust approach to service redesign specifically tailored for the provision of MH care and services to CYP consumers and their families experiencing acute MH problems; (2) data to support an economic evaluation to determine the cost-effectiveness of the Safeguards program compared with treatment as usual to inform future decision-making; (3) a mixed methods approach to explore any improvements regarding engagement, access, and uptake/integration of care in mental MH with the inclusion of the Safeguards Teams within the CAMHS as well as clinical practice changes within ED, MH services and community-based settings to prevent or decrease avoidable hospital ED presentations and hospital admissions (i.e., ED avoidance, facilitated linkage and engagement with services).

Moreover, the Safeguards Program will draw on building connections with local stakeholders and improving service delivery across CYP. As a result of embedding the new Safeguards Teams into the existing CAMHS structures, it will also ensure that access to MH care is based on level of acuity, risk, complexity, and distress, and is not limited by diagnosis or co-morbidities.

## (6) Conclusion

Outcomes associated with the impact, implementation, and economic evaluation of the STP are expected to assist with identifying gaps in acute care for children and adolescents in the community and provide an alternative model of care for CYP presenting in MH crisis.
